# Negative gas adsorption transitions and pressure amplification phenomena in porous frameworks

**DOI:** 10.1039/d4cs00555d

**Published:** 2025-01-27

**Authors:** Simon Krause, Jack D. Evans, Volodymyr Bon, Irena Senkovska, François-Xavier Coudert, Gulliaume Maurin, Eike Brunner, Philip L. Llewellyn, Stefan Kaskel

**Affiliations:** a Nanochemistry Department, Max-Planck-Institute for Solid State Research 70569 Stuttgart Germany s.krause@fkf.mpg.de; b School of Physics, Chemistry and Earth Sciences, The University of Adelaide South Australia 5000 Australia; c Faculty of Chemistry and Food Chemistry, TU Dresden Bergstrasse 66 01062 Dresden Germany volodymyr.bon@tu-dresden.de stefan.kaskel@tu-dresden.de; d Chimie ParisTech, PSL Research University, CNRS, Institut de Recherche de Chimie Paris 75005 Paris France; e Institut Charles Gerhardt Montpellier UMR 5253 Univ. Montpellier CNRS UM ENSCM, Université de Montpellier, Place Eugène Bataillon 34095 Montpellier cedex 05 France; f Institut Universitaire de France (IUF) France; g Aix-Marseille Univ., CNRS, MADIREL (UMR 7246) 13013 Marseille France

## Abstract

Nanoporous solids offer a wide range of functionalities for industrial, environmental, and energy applications. However, only a limited number of porous materials are responsive, *i.e.* the nanopore dynamically alters its size and shape in response to external stimuli such as temperature, pressure, light or the presence of specific molecular stimuli adsorbed inside the voids deforming the framework. Adsorption-induced structural deformation of porous solids can result in unique counterintuitive phenomena. Negative gas adsorption (NGA) is such a phenomenon which describes the spontaneous release of gas from an “overloaded” nanoporous solid *via* adsorption-induced structural contraction leading to total pressure amplification (PA) in a closed system. Such pressure amplifying materials may open new avenues for pneumatic system engineering, robotics, damping, or micromechanical actuators. In this review we illustrate the discovery of NGA in DUT-49, a mesoporous metal–organic framework (MOF), and the subsequent examination of conditions for its observation leading to a rationalization of the phenomenon. We outline the development of decisive experimental and theoretical methods required to establish the mechanism of NGA and derive key criteria for observing NGA in other porous solids. We demonstrate the application of these design principles in a series of DUT-49-related model compounds of which several also exhibit NGA. Furthermore, we provide an outlook towards applying NGA as a pressure amplification material and discuss possibilities to discover novel NGA materials and other counterintuitive adsorption phenomena in porous solids in the future.

## Abstract 2

The discovery of Negative Gas Adsorption (NGA), its experimental characterization, and the toolbox for systematic understanding are reviewed, along with an outlook on future applications and materials discovery pathways.

## Graphical abstract

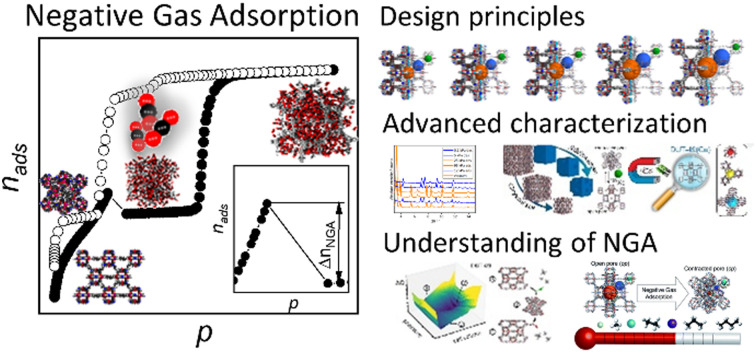

## 1. Introduction

Adsorption technology plays a key role in the separation industry, replacing energy intensive distillation processes.^1^ In recent years, environmental technologies, air capture, CO_2_ sequestration and gas storage have been advanced through innovative porous adsorbents.^2–6^ Novel porous materials play a key role in advancing these technologies and an understanding of the underlying mechanism is crucial for future developments. While the majority of porous materials are considered to be rigid, because they only undergo minor structural changes upon adsorption of gases and liquids, in recent years an increasing number of soft porous crystals (SPCs) have been discovered that can significantly change their pore structure in terms of size and shape in response to a stimulus.^7^ In many cases, this stimulus is of molecular origin (adsorption of gas, vapor, and liquid) but light and electric field can also induce structural deformations.^8–10^ Many SPCs exhibit expansion or contraction of the porous structure *via* adsorption-induced stress. As a result of the adaptive transformation of SPCs, their adsorption isotherms often exhibit unconventional shapes and steps.^11^ The dynamic pore space adaption and cooperative adsorption^12^ imply technological advantages, such as an ideal working capacity and internal heat management in gas storage,^3,13^ increased selectivity in separation,^6,14^ including isotope separation^4,5,15^ or new applications as sensors^16,17^ and actuators.^18^

**Fig. 1:**
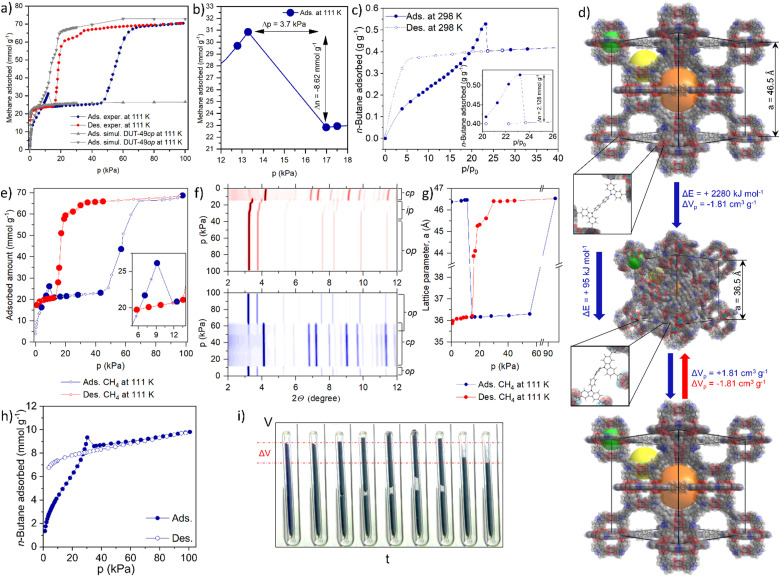
Negative gas adsorption transitions in DUT-49: (a) volumetric methane physisorption on DUT-49(Cu) at 111 K and GCMC simulated isotherms for DUT-49 op and cp phases; (b) NGA range of the adsorption isotherm indicating the expelled amount and pressure amplification in the cell; (c) gravimetrically measured adsorption of *n*-butane on DUT-49(Cu) at 298 K; (d) crystal structures of DUT-49op and DUT-49cp and the mechanism of the structural contraction and reopening; (e) volumetrically measured methane adsorption/desorption on DUT-49(Cu) at 111 K (large circles identify the points in which PXRD is measured) and (f) PXRD patterns, measured in selected points of the adsorption (bottom, blue contour) and desorption (top, red contour) isotherm; (g) evolution of the unit cell parameter *a* upon adsorption and desorption of methane at 111 K; (h) volumetrically measured adsorption and desorption of *n*-butane on DUT-49(Cu) at 298 K; (j) macroscopic changes of the adsorption bed upon *n*-butane physisorption at 298 K (the figure is adapted from ref. 19. Copyright Springer Nature 2016).

Gas adsorption processes are frequently studied using isothermal adsorption experiments *i.e.* adsorption isotherms displaying the amount of gas adsorbed as a function of the pressure in the gas phase. However, for all porous materials a mutual motif is a non-negative slope of the adsorption isotherm *i.e.* the adsorbed amount monotonically increases with increasing pressure (activity) of the adsorptive. The monotonic function in single component isotherms is a consequence of equilibrium thermodynamics. In 2015 we discovered the first adsorption isotherm seemingly violating this general expectation.^19^ A negative step was observed in the methane adsorption isotherm at 111 K of the metal–organic framework DUT-49 (49th material made at Dresden University of Technology) demonstrating a drop in uptake associated with a sudden gas-release from the framework after reaching a critical pressure. By dosing gas to the sample container reaching a characteristic pressure (*p*_NGA_), instead of further adsorbing, the material responds with gas desorption by releasing gas molecules (Δ*n*_NGA_, moles of gas desorbed per gram of MOF at *p*_NGA_) from its pores (Fig. 1) causing an overall pressure increase in a closed sample volume surpassing the initial dosing pressure, a phenomenon termed pressure amplification (PA). This counterintuitive phenomenon led to a new class of pressure amplifying materials as a result of negative gas adsorption transitions (NGA).

In this account we describe the discovery and mechanism of NGA and guiding principles for the design of pressure amplifying materials. We demonstrate *in situ* analytical methods *i.e.* parallelized adsorption/diffraction or adsorption/spectroscopy techniques in combination with complementary *in silico* simulations to be essential for achieving an in-depth understanding of the underlying mechanisms affecting NGA. We demonstrate systematic tailoring of new porous model materials with predefined micromechanical properties leading to an in-depth understanding of the framework structure and composition, adsorption conditions, as well as effects of crystal size and defects affecting NGA. As an outlook we discuss the possibility to apply NGA as a process of pressure amplification and illustrate approaches to discover novel NGA materials and other counterintuitive adsorption phenomena.

## 2. Discovery and basic mechanistic understanding

In 2012 we reported DUT-49 as a new benchmark and record material for methane storage applications at ambient temperature.^21^ The framework of DUT-49 is composed of interconnected metal–organic polyhedra (MOPs) built from the tetra-connective linker 9,9′-([1,1′-biphenyl]-4,4′-diyl)bis(9*H*-carbazole-3,6-dicarboxylic acid (H_4_bbcdc) and copper(ii) dimers. DUT-49 crystallizes in the cubic space group *Fm*3̄*m* and the ordered arrangement of the MOPs (*d*_p_ = 1 nm) is found to reflect a cubic close packing in **fcu** topology with open tetrahedral (*d*_p_ = 1.7 nm) and octahedral (*d*_p_ = 2.4 nm) voids (*d*_p_ - pore diameter). After activation with supercritical CO_2_, the nitrogen adsorption isotherm at 77 K showed a high uptake of 79 mmol g^−1^ from which an apparent specific BET area of over 5460 m^2^ g^−1^ and specific pore volume of 2.9 cm^3^ g^−1^ were derived. Gravimetric adsorption experiments demonstrated a high methane storage capacity of 308 g kg^−1^ (excess) at 11 MPa and 298 K, the highest reported gravimetric storage capacity for methane in any porous material at that time.^21^ This outstanding methane storage ability motivated us to study the methane adsorption behavior of DUT-49 in depth and we recorded isotherms at lower temperature, in particular the methane standard boiling point (111 K). Surprisingly, methane adsorption isotherms recorded at 111 K exhibit an untypical shape and contain a spike and drop in uptake with increasing pressure in the pressure region of 10–15 kPa^5^ (Fig. 1a and b).

With a magnitude of Δ*n*_NGA_ = 8.62 mmol g^−1^ the drop in adsorbed amount is comparable to the total adsorption capacity of commercial microporous adsorbents such as activated carbon and zeolites, and is unprecedented in the literature. As the low temperature conditions were quite unusual, in particular with respect to general adsorption applications, we were curious to find adsorptives that could stimulate a similar behavior at room temperature. To our surprise, adsorption experiments with *n*-butane at 298 K showed similar NGA transitions at the pressure of *ca*. 30 kPa.^19^ After presenting these unique and counterintuitive findings at topical conferences, world renowned experts questioned our findings and recommended further confirmation by use of various manometric and gravimetric adsorption instruments (Fig. 1c and h). These techniques confirmed the generality of our findings.

Today one can reproducibly observe NGA transitions in any laboratory. An instructive demonstration is to visually follow the process by filming a sample of DUT-49 in a capillary with a closed bottom during *n*-butane adsorption at 298 K (Fig. 1i). When reaching the critical pressure, the sample moves upwards due to the gas release and subsequently the sample bed contracts by *ca.* 20%. Such macroscopic structural transformations are of microscopic origin, caused by colossal dynamic structural transformations of the porous framework. As an ultimate confirmation we recorded powder X-ray diffraction (PXRD) patterns *in situ* in parallel to the adsorption process of both *n*-butane at 298 K and methane at 111 K (Fig. 1e and f). The small differences in *p*_NGA_ observed in *ex situ vs. in situ* experiments in the case of methane (Fig. 1a, b and e) and volumetric *vs.* gravimetric physisorption of *n*-butane (Fig. 1c and h) can be explained by the uncertainty in the thermometer calibration of the corresponding setups. In both cases, we could identify a transition of the crystal structure of DUT-49 after the NGA step to a previously unknown structure. By refining the obtained diffraction patterns we could identify that the original unit cell volume of the open pore state (op) of DUT-49 (*Fm*3̄*m*, *a* = 46.427(4) Å) contracts by *ca.* 50% (Fig. 1g). Only at higher pressure (>60 kPa for methane at 111 K) the cp (cp - contracted pore) framework (*Pa*3̄, *a* = 36.1603(2) Å) was found to reopen to the op state, indicating that the cp state is only stable in a certain intermediate pressure range (Fig. 1d). Upon desorption the op phase transforms into intermediate phases (ip) (*Pa*3̄, *a* = 45.542(2) Å) and, at pressures below 15 kPa (methane 111 K), irreversibly into the cp phase.

Rietveld analysis of the cp phase revealed that the original mesopores in the op structure (>2 nm) are contracted to less than 1 nm in diameter reducing the overall pore volume by over 70% explaining the pushout of the gas due to a reduction in pore volume. Excess methane that no longer fits into the pores is simply squeezed out from the porous framework and released back into the gas phase resulting in a stimulated pressure amplification. The amount of gas released in the NGA step, Δ*n*_NGA_, is thus defined as the difference in amount adsorbed in the op phase before contraction, *n*_ads,op_, and in the cp phase after contraction, *n*_ads,cp_.

1 Δ*n*_NGA_ = *n*_ads,op_ − *n*_ads,cp_

These characteristic features of NGA are based on long-lived metastable states along the adsorption isotherm. Pressure amplification is a transient process resulting from NGA in a closed system that can be monitored as a function of time.

*In situ* EXAFS studies revealed that the Cu(ii)-paddle wheel nodes forming the MOPs do not significantly distort during that transformation, however, the biphenyl units, bridging adjacent MOPs, undergo a buckling deformation (Fig. 1d). The intactness of the paddle-wheel (PW) clusters was later also confirmed by EPR spectroscopy in the presence of *n*-butane (298 K), diethyl ether (298 K), xenon (156 K), and ethylene (165 K) indicating a process that relies on the change in pore volume and not in the alteration of specific adsorption sites.^22,23^ This comprehensive structural analysis provides a good explanation for the NGA process, but what driving forces are responsible for the contraction that is strong enough to even increase the outer gas pressure?

Multi-level *in silico* simulations were crucial to rationalize NGA.^24^ In fact, DUT-49 is not the only framework that undergoes structural contraction upon gas adsorption. The op–cp–op trajectory (with increasing pressure along the isotherm) is typically classified as the breathing behavior and was first reported in MIL-53 with a unit cell volume contraction of 50%.^11,25^ It implies that the guest-free op state has a lower Helmholtz free energy than the guest-free cp form (*F*_op_ < *F*_cp_). This is intuitive when comparing the conformation of the highly strained (buckled) biphenyl unit in the cp form of DUT-49 to the quite regular and highly symmetric conformation in the op-framework (Fig. 1d). Simulations and experimental calorimetric validations confirm the absolute amount of adsorption enthalpy for methane in DUT-49cp (|Δ*H*_ads,cp_| = |*n*_ads_·Δ_ads_*h*_cp_|) to be 50% larger compared to that of DUT-49op for an intermediate loading. This overall exothermic energetic gain is the driving force for the structural contraction. This finding is quite intuitive as the molar adsorption enthalpy generally increases with decreasing pore size (|Δ_ads_*h*_cp_| > |Δ_ads_*h*_op_|) due to enhanced confinement effects and pore–wall interactions. At intermediate loading and pressure |*n*_ads_·Δ_ads_*h*_cp_| exceeds |*n*_ads_·Δ_ads_*h*_op_| stabilizing the cp form, but at high pressure the op form can adsorb more molecules (*n*_ads,op_ ≫ *n*_ads,cp_) and hence the op form is the thermodynamic minimum (|Δ*H*_ads,op_| > |Δ*H*_ads,cp_|). This thermodynamic evolution rationalizes breathing in microporous MOFs such as MIL-53^20^ and is transferable to mesoporous MOFs such as DUT-49. But why does MIL-53 not show any NGA transitions?

**Scheme 1:**
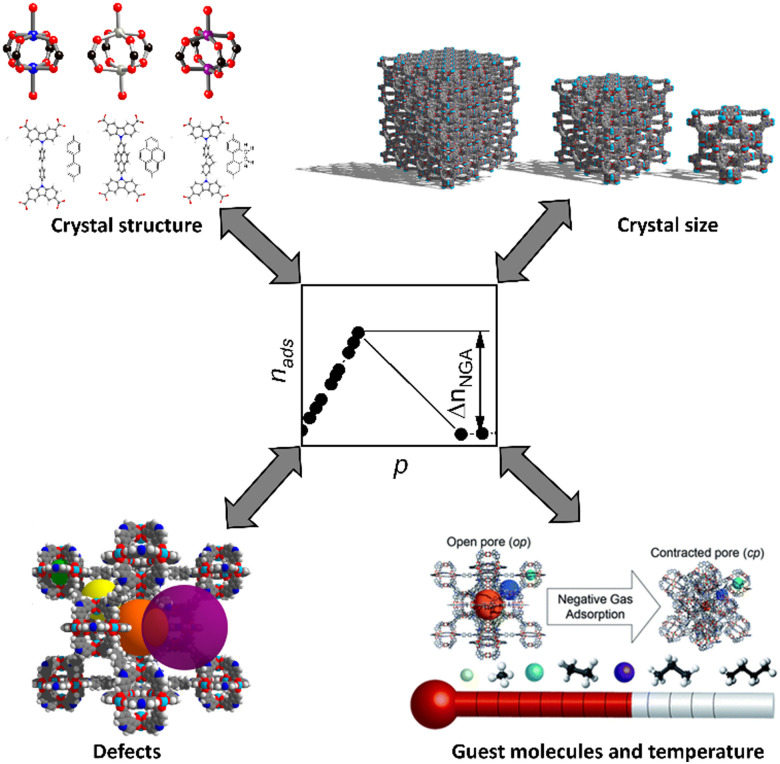
Major factors influencing NGA (the figure is adapted from ref. 27–30).

The characteristic origin of the counterintuitive NGA transition is the *metastability* of the overloaded op-form surpassing the equilibrium transition pressure at which isotherms of the op and cp form intersect leading to a transition occurring far from equilibrium and the associated counterintuitive gas release. Such long-lived metastable states are caused by energetic barriers that cannot be overcome by thermal system fluctuations.^11^ Stimulated by such counterintuitive observations in SPCs, advanced simulations were further developed allowing inclusion of these energetic barriers by using hybrid methods which combine Monte Carlo and molecular dynamics simulations (see the section on computational models for details).^26^ Such simulations cover the full energetic landscape of the framework with respect to changes in the unit cell dimensions, the gas pressure, and the guest loading at defined temperatures.^26^ While the trajectory along the energetic minima generates a hypothetical equilibrium isotherm that contains structural contraction and reopening upon methane adsorption at 120 K, hysteresis and NGA are observed when considering an energetic barrier of 15 *k*_B_*T*. Consequently, we can derive: (i) along the adsorption branch, at the intersection of the op and cp isotherm the barrier cannot be overcome and the system retains an overloaded op state with increasing pressure/gas loading before the transition to a cp form occurs releasing the excess of gas that exceeds the capacity of the cp phase; (ii) along the desorption branch, the op-form first transforms into ip phases and ultimately into the cp state and remains in this structure even after evacuation of the system for several days. Hence, after one adsorption–desorption cycle DUT-49 does not retain the op form because the barrier for reopening upon desorption is too large. Experimentally, NGA can be repeated multiple times on the same sample by triggering structural reopening to the op phase at the adsorption temperature, followed by gentle desorption of the guests under supercritical conditions. Despite advanced simulations reproducing experimental isotherms without input from experimental findings, the effective barrier imposed in the simulations is a generic system barrier arbitrarily set at a value in the range of 0–25*k*_B_*T*.^24,26^ This approach does not identify the microscopic barriers origin in particular bridging length scales from the molecular to the single crystal level. Energetic barriers are typical features of first order phase transitions, associated with nucleation. For NGA transitions these nucleation barriers are intrinsic to the solid phase transformation (large unit cell volume changes) as well as fluid nucleation phenomena (phase transitions of the fluid in the pore). Hence, the predominant factors affecting NGA are (i) the solid material structure including non-idealized structure effects, (ii) the gas probe molecule and its characteristics, and (iii) external variables (*p*,*T*) governing the phase diagram and adsorption thermodynamics (Scheme 1).

In the following we outline these three factors governing NGA using a model-material approach *i.e.* we synthesized defined model materials allowing us to test hypotheses explaining the origin and critical aspects of NGA supported by advanced simulations and *in situ* characterization methods.

## 3. The role of framework structure: guidelines for PA materials

### 3.1. Primary structure of frameworks: pore size, linker, node, topology, *etc.*

Designing model materials for the rationalization of novel physical phenomena is an emerging art in materials science.^31^ Bistability as explained above is a prerequisite for breathing frameworks. But what factors are governing metastability of the solid? Is Δ*n*_NGA_ an appropriate measure to quantify metastability? In the following we first consider the ideal periodic crystal structure (the constituents and framework architecture) as the prime vector for tailoring PA materials.

**Fig. 2:**
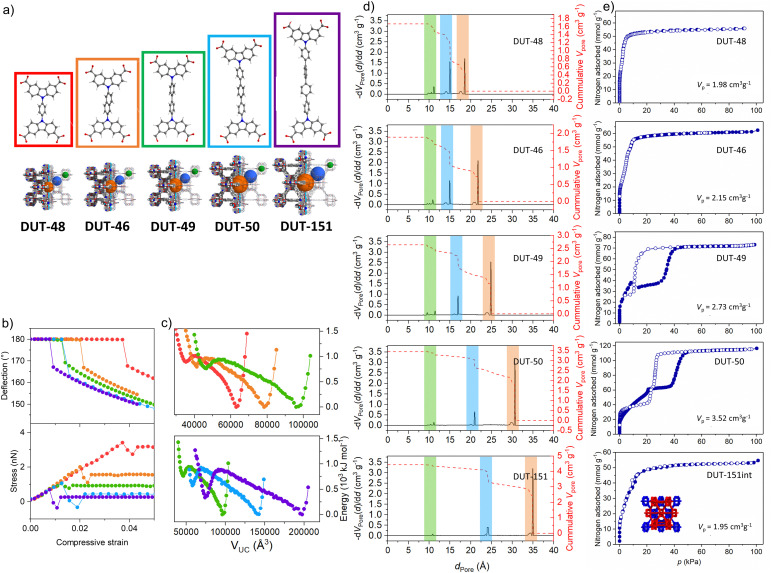
Ligand elongation strategy in the isoreticular series of DUT-49(Cu) for the fine control over mechanical stress: (a) ligand structures and resulting frameworks; (b) strain–stress analysis of the linker molecules; (c) free energy profiles calculated for the isoreticular series showing two distinct minima for each MOF; (d) pore size distributions for the isoreticular series of DUT-49-related MOFs; (e) nitrogen physisorption at 77 K measured on the DUT-49 and related frameworks (the figure is adapted from ref. 32. Open access Springer 2019).

In porous material design, the first obvious question is: what is the role of pore size and geometry for NGA transitions? To tackle this question, we synthesized a whole series of DUT-49 isoreticular frameworks.^32^ By systematically varying the spacing between adjacent MOPs using phenylene (DUT-48), naphthalene (DUT-46), biphenylene (DUT-49), triphenylene (DUT-50) and tetraphenylene (DUT-151) as struts the pore sizes could be systematically expanded from 1.85 (DUT-48) to 3.07 nm (DUT-50, octahedral pore) within the same framework topology (Fig. 2a). DUT-151 was synthetically obtained as a doubly interpenetrated framework structure (DUT-151-int) with **fcu-a** topology. Hence, DUT-151-int is not considered further here, as simulations demonstrated that interpenetration suppresses the structural transformation required for NGA leading to a different type of flexibility (framework displacement) (Fig. 2e).

While DUT-48 and 46 with the smaller pore sizes show no breathing in methane adsorption isotherms at 111 K and typical type I isotherms, DUT-49 and -50 demonstrate breathing and distinct NGA transitions (Fig. 2e). DUT-50 was the second framework identified as a pressure amplifying material indicating that larger pores (>2 nm) support NGA. But an important aspect is also the difference in porosity of op and cp forms as this accessible pore volume change defines the amounts adsorbed (*n*_ads,op_, *n*_ads,cp_) and the maximal achievable Δ*n*_NGA_ (eqn (1)). However, the pore size and micromechanics of the framework are interconnected, as the strut elongation reduces the force required to buckle the ligand (see below). Frameworks with smaller pores are stiffer than large-pore networks and a higher adsorption stress is required to buckle the shorter struts (Fig. 2b and c). Hence, a framework appearing rigid when exposed to a weakly interacting guest (N_2_, CH_4_) may deform upon exposure to a strongly interacting guest (CO_2_).^34^ When comparing the primary structural effects in model materials one should therefore consider the change in the driving stimulus or try to render it as a constant. A generic slit-pore model confirms the subtle interplay of structural micromechanics and pore size to be decisive for finding a parameter window allowing NGA to occur.^35^ However, this model is structurally featureless and does not provide a chemical intuition for the design of new real-world NGA materials beyond DUT-49 and its derivatives. In the following, it should be kept in mind that breathing is a necessary but not sufficient prerequisite to observe NGA.

#### 3.1.1. Linker

Beyond extended simulations, for an illustrative and chemically intuitive rationalization of the role of the linker backbone one can compare the strut connecting the MOPs to the buckling of a column, as described by Euler's equation:2
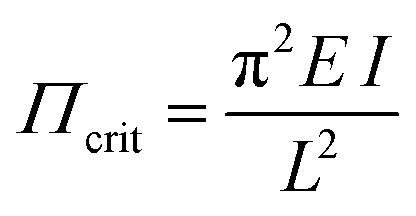


The critical tension *Π*_crit_ leading to buckling decreases with increasing length *L* of the strut (keeping *E* and *I* constant; *E* = elastic modulus, *I* = moment of inertia).

**Scheme 2:**
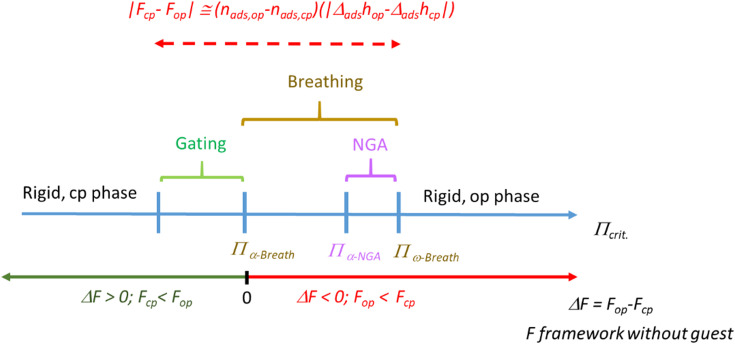
Rationalization of the NGA regime with the view of primary structural deformability.

This formula nicely illustrates the effects of linker elongation in the model materials series DUT-48, -46, -49 and -50, leading to a softening of the framework with increasing *L*. In other words, for a given adsorptive there will be a critical length required to allow the adsorption stress to achieve the buckling as a prerequisite for breathing and NGA. But, as pore size and framework stiffness are simultaneously altered it is impossible to delineate the sole effect of linker stiffness as the origin of NGA. For analyzing the sole impact of the ligand elasticity, while keeping *L* constant, we synthesized a second series of model frameworks.^30^ Replacing the biphenyl strut with a pyrene backbone (DUT-147) the framework is rigidified, and breathing is suppressed (for *n*-butane, 298 K). *n*-Butane has a higher |Δ_ads_*h*| and represents a stronger interacting guest than methane (see below). In contrast, introducing an ethylene sidegroup in the backbone (DUT-148) does not lead to NGA suppression for the same guest. Although the pore size is affected slightly in these modifications, a rationalization based on Euler's formula is obvious as with increasing *E* and *I* the critical pressure required for buckling increases. We can propose several regimes for a given guest. Essentially the range *Π*_α-Breath_ to *Π*_ω-Breath_ characterizes the regime in which breathing can be observed because the empty open framework is more stable than the cp phase and Δ*F* is of the same order as the adsorptive interaction (*n*_ads,op_ − *n*_ads,cp_) multiplied with |Δ_ads_*h*_op_ − Δ_ads_*h*_cp_|) (Scheme 2, *Π* stands here for the critical micromechanical buckling tension as a measure for framework deformability, in simple terms it stands for “framework stiffness”). Above a characteristic *Π*_ω-Breath_ the framework remains in the op phase and does not contract at any loading because the framework is too stiff, and below *Π*_α-Breath_ the strut is so soft that the cp phase is stabilized *vs.* op (*F*_cp_ < *F*_op_), potentially leading to a gating type characteristic. However, a certain intermediate stiffness (characterized here by *Π*_α-NGA_) is required to suppress the contraction when the equilibrium transformation pressure is reached leading to the “overloaded metastable adsorptive-framework complex” (guest@DUT-49op*) and a structural barrier the stimulus has to surpass. Hence, from a microstructural view of the framework stiffness a characteristic range from *Π*_α-NGA_ to *Π*_ω-Breath_ is expected for a specific framework topology and guest leading to NGA transitions.

#### 3.1.2. Metal node

The paddle wheel nodes also play a certain role in the micromechanics of the systems. In general DUT-49(M) is obtained with several PW forming metals (M = Zn, Cd, Ni, Cu, Co, Mn, *etc.*).^36^ However, only DUT-49(Cu) and DUT-49(Ni) could be desolvated without pore collapse (*i.e.* amorphization and loss of porosity). Even supercritical drying and careful handling under dry conditions could not prevent the pore collapse. Zn^2+^-complexes may easily change their coordination geometry. In several framework systems containing Zn_2_-paddle wheels this leads to softening stabilizing the cp-phases *vs.* op-phases.^37^ To date it is not entirely clear at which stage the framework collapses, whether during exchange of the solvent by CO_2_ or during sc-CO_2_ desorption. The softness of Zn-based frameworks may even under mild CO_2_ desorption conditions lead to a deformation as the desorption stress is high for this probe molecule. It may well be that supercritical argon drying will preserve the op framework, but this has not been explored until today. In order to shine light on the mechanism of solvent desorption in DUT-49(M), the desolvation of the MOFs in a nitrogen flow was followed by time-resolved synchrotron PXRD indicating continuous op → ip transition in the case of DUT-49(Cu), DUT-49(Ni) and DUT-49(Zn).^38^ Subsequently a first order transition ip → cp was observed for DUT-49(Cu) and DUT-49(Ni). Direct amorphization was observed for all other frameworks indicating that ip and cp phases for all other metals cannot be stabilized, and that even the guest free op phase is not thermodynamically stable. These observations follow the trends of the Irving–Williams series predicting the highest stability for Cu(ii) and Ni(ii) paddle-wheel-type metal complexes.^39^

So far only Cu^2+^ and Ni^2+^- based DUT-49 frameworks could be fully activated retaining crystallinity. The higher strain energy required to transform Cu_2_- and Ni_2_-PWs has also been rationalized by density functional theory (DFT) calculations.^33^ Although one should not overemphasize the role of magnetic interactions, as the energetics are inferior compared to host–guest interactions, the situation may become quite complicated. The Cu-PWs show a typical antiferromagnetic coupling with a magnetic *S* = 0 ground state <80 K.^22,23,40^ EPR spectroscopy gives important insights and is a sensitive technique also used to analyze the coordination of axially bound donor molecules. As the antiferromagnetic coupling and axial bonds affect the stiffness of the PW, a subtle temperature and guest dependent impact on the node flexibility should also be considered. However, these minor effects are not accurately enough captured by advanced theoretical methods and more studies are required. Hence, NGA has so far only been observed for Cu_2_-PW-based frameworks and not for any other metal. Given the little deformation of the metal-node upon contraction in DUT-49 and derivatives, the pronounced stability of the nodes plays an important role for the intactness of the MOPs constituting the framework. The ability to coordinate additional donor molecules at the open metal sites leads to significant differences for certain adsorbates (for example CO *vs.* N_2_) affecting the NGA behavior discussed below.^41^ However, current understanding does not provide indications to assign peculiar magnetic, structural or reactivity features of Cu_2_-PWs as an origin of NGA beyond the mere role of mechanical stabilization of the framework. *In situ* diffuse reflectance infrared Fourier transform (DRIFT) spectroscopy in parallel to the adsorption of *n*-butane at 295 K in DUT-48, -46, -49, and -50 shows little change to the bands associated with the carboxylate modes reflecting the intactness of the coordination motif in the framework. However, significant changes in the spectra (in particular modes corresponding to C–C and C–N vibrations of the ligand backbone) are observed for only DUT-49 and -50 that undergo structural transitions upon *n*-butane adsorption, strongly supporting that the molecular mechanism of structural contraction is based on the deformation of the ligand rather than the metal node.

#### 3.1.3. Framework topology

So far, NGA has only been reported for DUT-49 analogous frameworks in the **fcu** topology. It remains an open question, whether other topologies may also lead to NGA transitions.

A general theoretical approach assuming a bistable slit pore model and generic forces (barriers) for the op–cp transformation predicted NGA to be a general phenomenon independent of pore shape and topology.^35^ However, a critical ratio of pore size, adsorption stress, and framework rigidity opens only a relatively narrow window of parameters where NGA can be observed. The search for new NGA materials has just begun! But which structural space beyond DUT-49 would be best to search for?

We suspected DUT-13 to be a good candidate as we had already observed quite unexpected adsorption isotherms for this framework with a corundum-like hexagonal (**cor-a** or **ttu-a)** topology.^42^ Zn_4_O-clusters are connected by BenzTB *N*,*N*,*N*′,*N*′-benzidine tetrabenzoate linkers to form a framework with high porosity. In fact, this framework showed a similar breathing behavior in methane adsorption isotherms, indicating bistability which was in the first publication not understood due to the lack of *in situ* instrumentation. A subsequent deeper analysis of the structural changes revealed the significant deformation of the framework and breathing in a temperature range from 111 K to *ca.* 140 K without NGA.^43^ The lack of NGA in this case could be due to the smaller pore size (*ca.* 1.8 nm), a lower barrier for the framework transformation, or the topology. DUT-13 is highly anisotropic and the hexagonal system has elongated elliptical pores (1.8 × 2.9 nm). The linker lacks the bond connecting the aromatic rings which leads to a larger opening angle in the DUT-13 linker (120°) compared with the DUT-49 linker (90°) and a higher degree of conformational flexibility. Such softening may favor a transformation without metastable states (*Π*_crit_ < *Π*_α-NGA_) and a reduced activation barrier. An extended version of DUT-13 is DUT-190 leading overall to a larger pore size (1.8 × 3.8 nm), but the channels are elongated and the width remains below 2 nm.^44^ DUT-190 breathes akin to DUT-13 but without NGA transitions for gases such as nitrogen (77 K), methane (111 K), carbon dioxide (195 K) and xenon (200 K). *In situ* PXRD data, collected in parallel to nitrogen physisorption at 77 K, indicate amorphous contracted phases forming as intermediates with further reopening to the op phase at higher pressures. This lack of defined crystalline cp-phase may be another reason for the lack of NGA despite the high porosity and bistability. On the other hand, reduced conformational freedom by introducing a C2 handle and additional methyl groups in a similar linker (5,5′-(3,3′,5,5′-tetramethyl-[1,1′-biphenyl]-4,4′-diyl)bis(10,11-dihydro-5*H*-dibenzo[*b*,*f*]azepine-2,8-dicarboxylic acid) leads to a rigid framework, DUT-193 with a new topology (**bff**).^45^

Current knowledge indicates that topologies other than **fcu** with large enough pores should also lead to NGA but specific examples have neither been identified by simulation nor experimentally reported. This includes the very popular UiO-66 type materials, which are probably amongst the most stable MOFs known to date. An open question remaining to be answered is also whether the hierarchical pore structure (pore connectivity) of DUT-49 and the resulting pore filling mechanism plays a role for the transient metastable states leading to NGA as they lead to diverse hysteresis phenomena in other mesoporous MOFs, carbons, and silica.^46^ In principle a reduced diffusivity in proximity to the NGA transition could be an origin of hampered equilibration and overloaded states. However, pulse-field gradient (PFG) NMR studies in the presence of *n*-butane show exactly the opposite! A maximum in the diffusivity shortly before the op-cp transition occurs.^47^ Diffusion limitation in the interconnected pore system is certainly not the origin of NGA transitions.

### 3.2. Secondary structural characteristics: particle size and defects

The phase transitions induced by guest adsorption are cooperative phenomena that are strongly influenced by crystal size or, more precisely, by domain size effects.^48^ The crystal size dependent switchability of MOFs was reported in 2013 by Kitagawa and was termed “shape memory effect”. In recent years, size and shape dependent switchability has been investigated by several groups. A beautiful example was the synthesis of ZIF-8 nanoparticles varying in shape and size by Watanabe *et al.*^49^ Also, for DUT-8 the crystal size dependent switchability was studied in great detail.^50^

In some cases downsizing leads to a framework rigidification *e.g.* cooperative van der Waals interactions of linkers stabilizing the cp form are weakened leading to a relative stabilization of the op form.^48^ Moreover, the density of the adsorbed phase in the outer layer of the particles is reduced compared to the bulk reducing the guest–host interactions. Hence, in gating and breathing MOFs the op form is typically stabilized such that the breathing and gate closing transitions are suppressed or require a higher stress (adsorption or external) for the transformation to a cp form.^48^

For DUT-49, it was shown that below a characteristic particle size breathing and NGA transitions induced by N_2_ at 77 K are practically suppressed. Below 1 μm the solid behaves as a typical rigid porous material without any transformations during N_2_ adsorption. However, for adsorption conditions exerting stronger deformation stress (*n*-butane, 298 K) the op-cp transformations are retained but Δ*n*_NGA_ decreases with shrinking particle size. The latter may indicate that crystal size does not only affect Δ*F* = *F*_op_ − *F*_cp_ but also the energetic barrier. It can be expected that for a reduced number of unit cells this barrier vanishes, as we transit from a cooperative to a molecular structural change. So far there are only few simulations on finite size effects available, but the work by Schmid and Van Speybroeck and co-workers provides important insights into MOF nanoparticle transformation mechanisms.^51^ Nanosize effects in simulations and from thermodynamic calculations are expected in a size regime of 1–100 nm, several orders of magnitude lower than the ones observed in experimental studies (up to 1000 nm). This discrepancy indicates secondary effects (twinning, surface effects, defects, *etc.*) to superpose upon genuine nanosize effects.^48^ Model materials with narrow particle size distributions, uniform shapes, controlled defects *etc.* as well as advanced analytical techniques for real structure analysis may shine light on these discrepancies in the future. In DUT-49 it was found that only a high concentration of tailored structural defects can impact the NGA as investigated by PXRD and Xe-NMR.^28^

Despite these open questions, the findings are important and demonstrate crystal size and morphology to be a second “independent vector” massively impacting PA materials design. It cannot be emphasized enough that only comparable crystallite size framework structures can be compared in terms of idealized periodic structural modes (the primary structure discussed in section 3.1).

## 4. The role of the molecular guest stimulus

### 4.1. The role of guest molecules and temperature

As discussed above, adsorption-induced pore contraction is a prerequisite to observe NGA. Roughly speaking, the total gain in adsorption enthalpy upon structural contraction per unit cell, ΔΔ_ads_*H*_total_, at the intersection of the isotherms of the op and cp phase estimated by multiplying the difference in adsorption enthalpy in the op and cp phase, ΔΔ_ads_*h* = Δ_ads_*h*_cp_ − Δ_ads_*h*_op_ with the amount of gas adsorbed, *n*_ads_, has to exceed Δ*F* (a more precise theoretic analysis is discussed in the simulation section).^32^

3 Δ*F* < |ΔΔ_ads_*H*_total_|

4 ΔΔ_ads_*H*_total_ = Δ_ads_*h*_cp_·*n*_ads,cp_ − Δ_ads_*h*_op_·*n*_ads,op_

Hence, a guest with a high adsorption enthalpy (*e.g.* CO_2_, butane) can easily stimulate the contraction while guests with low adsorption enthalpy (*e.g.* H_2_) cannot exert enough contractive stress to achieve a deformation. This also implies that certain frameworks, for example DUT-46, appearing rigid while exposure to CH_4_ may become flexible upon exposure to a stronger interacting molecule like CO_2_.^52^

As the amount adsorbed (*n*_ads,cp_, *n*_ads,op_) for each guest is highly dependent on temperature it is obvious that for each guest above a critical temperature breathing (and hence NGA) vanishes. In other words the critical tension parameter *Π* in Scheme 2 should be viewed in relation to the adsorption stress.^53^

Breathing and the energetics discussed above are a necessary but not sufficient condition for NGA. Additionally, a barrier for the contraction has to delay the contraction. Beyond the energetic barriers arising from the framework architecture discussed above, observations indicate additional barriers arising from the phase transition of the guest. In mesoporous materials it has been known for a long time that nucleation phenomena of the fluid inside the mesoporous channels largely determine the adsorption/desorption hysteresis characteristic for mesoporous solids.^54,55^ In addition, the shape of the hysteresis can directly be linked to the adsorption-induced stress which reaches a contractive maximum upon capillary condensation.^56^ In this context it can be hypothesized that NGA is associated with mesopore filling processes and fluid nucleation barriers.

A detailed study of methane adsorption (111 K) using neutron diffraction and CD_4_ as the guest revealed indeed that the cuboctahedral pores of DUT-49 (*d* = 1.0 nm) are saturated before NGA. In combination with GCMC simulations we identified that NGA occurs in parallel with the filling of the medium (*d* = 1.7 nm) and in particular the largest pores (*d* = 2.4 nm) of the framework.^32^ The measurement of *n*-butane diffusivity with PFG NMR also showed that the diffusivity steadily increases with loading until the NGA step.^57^ More recently, a rigid variant of DUT-49 (DUT-149, details in the chapter on DUT-49 related materials) with almost the same pore size was analyzed *via* PFG NMR demonstrating a narrow hysteresis typical for mesoporous materials.^47^ Hence, DUT-49 and DUT-149 offer pore sizes at the onset of hysteresis observation (*n*-butane, 298 K) and nucleation barriers associated with nucleation and capillary condensation are the origin of metastability in these systems. These history dependent adsorption states are also evident from PFG NMR analysis of diffusivity in DUT-149 confirming the fluid nucleation barriers as an origin of NGA. Essentially the phase transition of the fluid is coupled to the solid phase transition of the framework and both transitions have their own activation barriers and complex *p*,*T*-dependence.

**Fig. 3:**
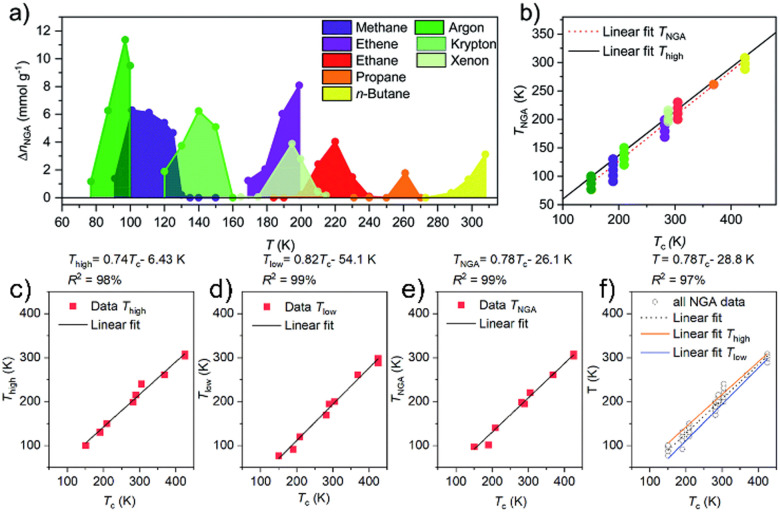
The role of temperature for breathing and NGA transitions in DUT-49(Cu): (a) Δ*n*_NGA_ for inert gases and hydrocarbons as a function of temperature; (b) linear correlation of Δ*n*_NGA_ and critical point of the fluids; (c)–(f) linear correlation of *T*_high_, *T*_low_, *T*_NGA_ and *T* average from the critical point (the figure is adapted from ref. 29 Copyright RSC 2021).

This view is corroborated by wide ranging analyses of a variety of guests stimulating NGA in DUT-49.^29^ Almost all permanent gases can induce NGA in DUT-49 (hydrocarbons, noble gases, *etc.*) but the absolute temperature range in which NGA is observed varies significantly. Also the amount of gas expelled (Δ*n*_NGA_) significantly depends on temperature and typically shows a non-linear characteristic when plotted against *T* (Fig. 3) with a maximum (Δ*n*_max,NGA_) at a characteristic temperature *T*_NGA_ for each gas investigated. The decrease of Δ*n*_NGA_ above *T*_NGA_ can be understood as *n*_ads_ decreases with higher *T* and hence the exothermic driving force ΔΔ_ads_*H*_total_ decreases. The latter can lead to incomplete contraction or the formation of ip phases as the adsorption induced stress decreases with increasing temperature. On the other hand the decrease of Δ*n*_NGA_ below *T*_NGA_ is akin to the exponentially increasing driving force of an undercooled phase transition.^29^

Interestingly *T*_NGA_ scales linearly with the critical temperature *T*_C_ of the investigated gases. This simple empirical correlation can be used to estimate the temperature range in which NGA is likely to occur for gases not investigated. This observation further indicates that the phase diagram of the fluid and the proximity to the boiling curve is a prerequisite to observe NGA. The nucleation of the liquid phase in mesoporous materials is affected by a barrier leading to long-lived metastable states similar to those in mesoporous silica materials.^55^ However, in DUT-49 this capillary condensation is coupled to a solid phase transition (inelastic transformation) with its own barrier for structural transformation.

Given that there is NGA in such a wide temperature range with different gases, the question arises whether temperature also has an effect on the structural contraction of the framework? DUT-49 shows a pronounced negative thermal expansion (NTE) coefficient of *α*_V_ = −32.8 MK^−1^ and *α*_L_ = −10.9 MK^−1^,^58^ which is comparable with a calculated value of *α*_V_ = −29.8 MK^−1^, but lower than calculated values for record holders MOF-399 (*α*_V_ = −87.6 MK^−1^) and NU-110 (*α*_V_ = −71.6 MK^−1^).^59^

Temperature dependent single crystal XRD studies conducted on the series of DUT-49(M) frameworks (M – Mn, Fe, Co, Ni, Cu, Zn, and Cd), containing DMF, ethanol and NMP in the pores, indicate NTE behavior above the melting point of the solvent. The freezing of the solvent induced the reversible contraction to an intermediate pore (ip) phase accompanied by 10% reduction of the unit cell volume. The analysis of the crystal structures of ip phases shed light on the mechanism of transition, as it causes in-plane bending of the linker.^58^

**Fig. 4:**
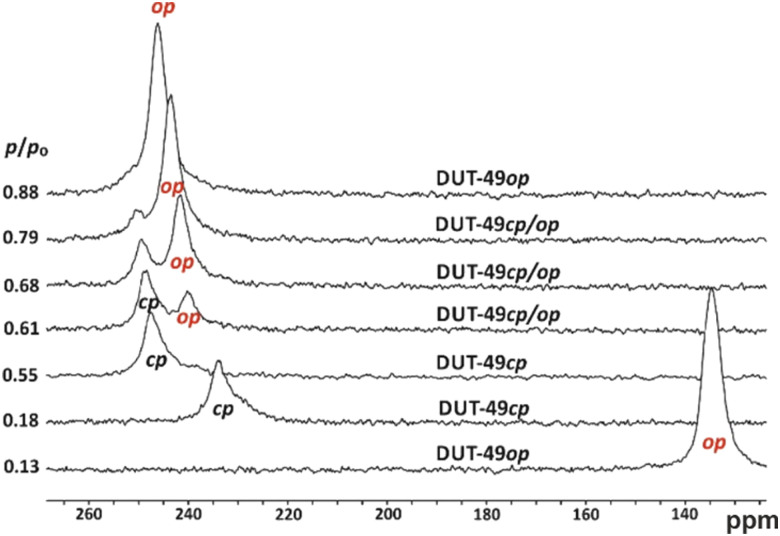
*In situ* ^129^Xe NMR spectra of DUT-49 measured at 200 K. Note the sudden chemical shift change by about 100 ppm in the narrow relative pressure range between 0.13 and 0.18. Reproduced and adapted with permission from ref. 61*b*. Copyright American Chemical Society.

Among the tested gases, Xe is a valuable probe for *in situ* NMR studies of NGA as it induces NGA and its chemical shift strongly depends on the pore size.^60^ Hence for Xe adsorption, the pore contraction is sensitively observed at 200 K by a sudden, tremendous shift from *δ* < 140 ppm (op) to *δ* > 230 ppm (*cf.*Fig. 4), while at higher pressure two peaks are observed indicating coexistence of the op and cp phases.^28,61^ Inside a crystal the Xe rapidly exchanges between all pores, and hence, only one peak reflecting the average pore size is observed. The emergence of a second peak is therefore clear proof of the simultaneous existence of cp and op crystals along the branch of reopening (cp − op) at higher *p*/*p*_0_. Two-dimensional NMR spectroscopy can estimate the exchange time of Xe atoms between the two pore systems (cp and op). The observed exchange time of 11 ms is characteristic for an inter-crystallite exchange. The coexistence of cp and op crystals which are not connected to each other but rather physically distant is characteristic of two phases (op, cp) that are not in equilibrium.

This indicates that the reopening is also an activated process and the steep 2nd adsorption step reflects the sequential opening of individual crystals. Hence, a phase coexistence in one crystal as postulated for MIL-53 *via* simulations can be ruled out for the breathing of DUT-49.^63^ It is, furthermore, remarkable that a high density of defects (*cf.* Section 3.2) results in the suppression of the NGA transition.^28^ This is then accompanied by the disappearance of this characteristic, a sudden chemical shift change described above for less defective DUT-49 (*cf.*Fig. 4).

The value of *in situ*^129^Xe NMR studies for the understanding of adsorption mechanisms is immense. At higher temperature (237 K) the system does not show the structural transformation in Xe adsorption and hence a monotonic change of the ^129^Xe NMR signal as a function of pressure, mostly reflecting the pore filling process and the density of Xe in the pore, is observed.

**Fig. 5:**
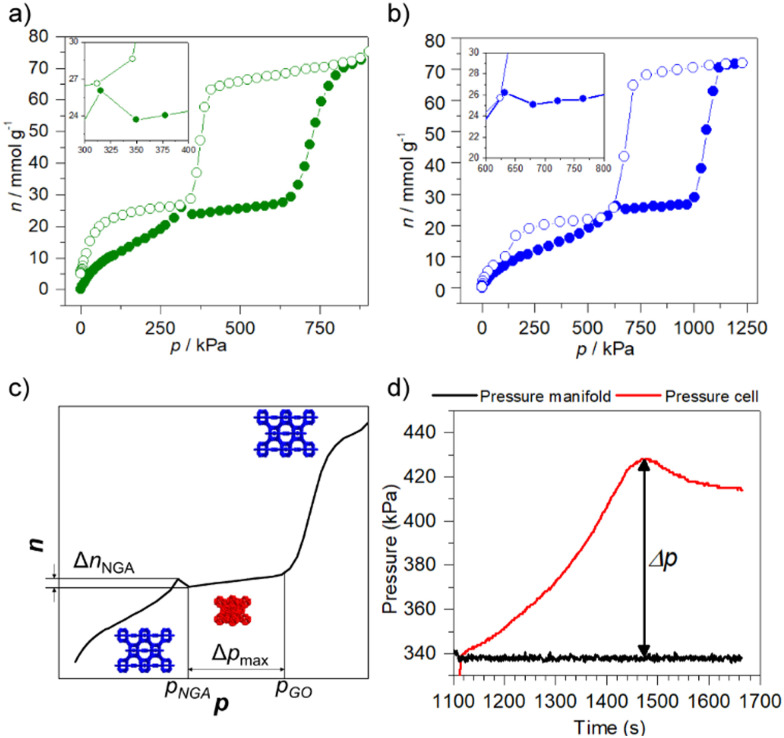
Physisorption of CO_2_ on DUT-49(Cu) and pressure amplification experiments: (a) physisorption of CO_2_ on DUT-49(Cu) at 230 K; (b) physisorption of CO_2_ on DUT-49(Cu) at 240K; (c) principle of the pressure amplifiers using the NGA effect in DUT-49(Cu); (d) pressure amplification experiment upon physisorption of CO_2_ on DUT-49(Cu) at 230 K (reproduced and adapted with permission from ref. 52. Copyright Wiley-VCH).

Hydrogen is a guest with very low adsorption enthalpy (typically |Δ_ads_*h*| = 5–6 kJ mol^−1^) and the difference in adsorption enthalpy upon contraction is consequently minor. Hence it is not surprising that DUT-49 does not contract upon hydrogen adsorption even at 20 K. CO_2_ on the other hand is a quite strongly interacting quadrupolar gas with a high adsorption enthalpy (|Δ_ads_*h*| = 50–120 kJ mol^−1^). The adsorption interactions of CO_2_ are strong enough to even contract DUT-46 (naphthalene strut) which is much stiffer and has a much higher *Π*_crit_. As the boiling pressure of CO_2_ at 230–240 K also extends into the high pressure range we were able to shift the *p*_NGA_ into the high pressure range demonstrating for the first time pressure amplification above atmospheric pressure (>300 kPa) (Fig. 5).^52^ The total pressure amplification also depends on the volume of the system. A minimum dead volume leads to the highest PA. DUT-49 achieves PA from 340 to 428 kPa using CO_2_ as the transmitting fluid, a quite substantial amplification demonstrating the power of PA as many pneumatic systems operate in that range.

**Fig. 6:**
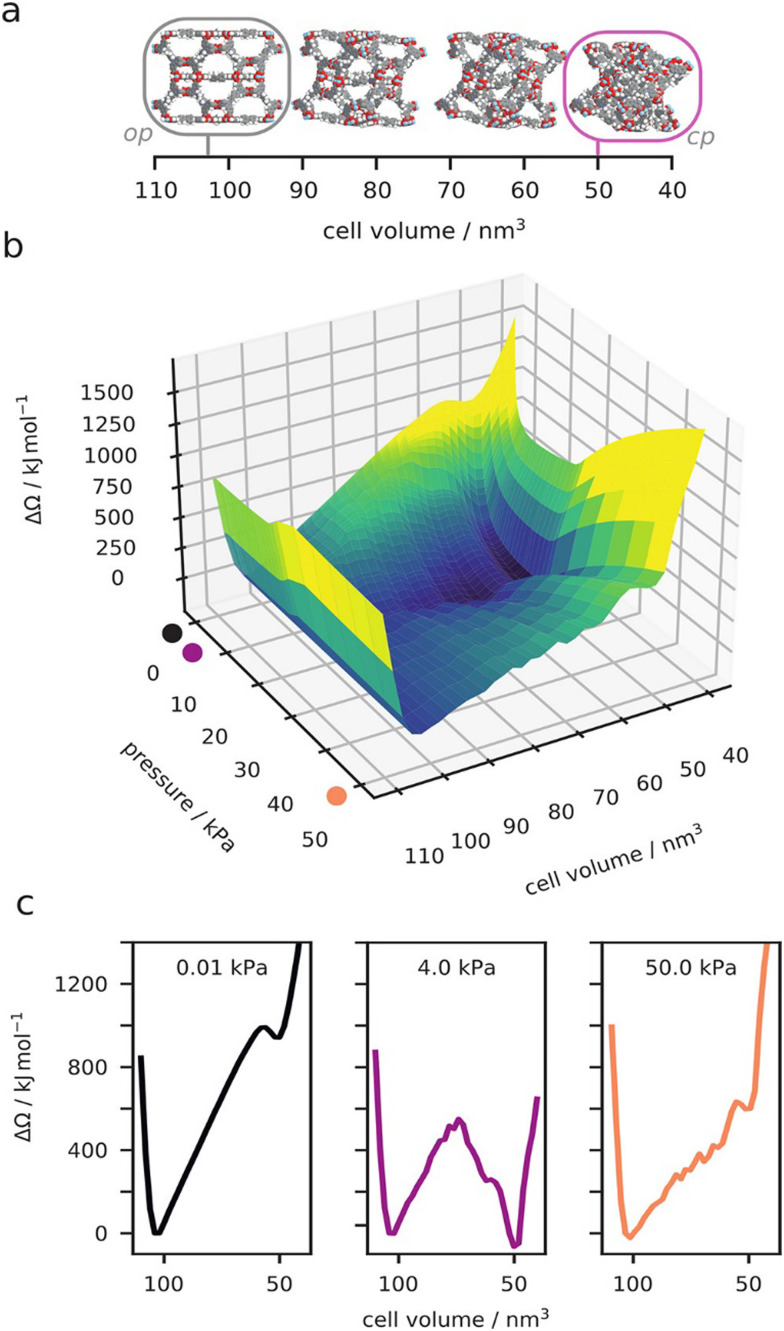
Computed osmotic surface of methane adsorption on DUT-49 at 120 K, as a function of unit cell volume (a) and methane gas pressure (b). Examples of the 1D osmotic surface at specific gas pressures (c). Reproduced and adapted with permission from ref. 26. Copyright American Chemical Society 2021.

Advanced calorimetry techniques give experimental access to adsorption enthalpies. Adsorption enthalpies were measured *in situ* for the op and cp form of DUT-49 proving indeed a higher interaction with DUT-49cp (Δ_ads_*h*_cp_ = −17 kJ mol^−1^) *vs.* DUT-49op (Δ_ads_*h*_cp_ = −10 kJ mol^−1^) at intermediate pressure.^41^ Significant differences in adsorption mechanisms were identified for CO *vs.* O_2_ and N_2_. CO chemisorbs to the paddle wheels and neither DUT-49 nor DUT-149 show NGA at 87 K. However, O_2_ induces NGA in DUT-147, 49 and 149 at 77 K but N_2_ only for DUT-49. These specific interactions in the case of CO demonstrate an intricate interplay of fluid–fluid *vs.* fluid–framework interaction that require more advanced simulation methods for rationalization in future but also indicate interesting opportunities to alter switchability by external molecular triggers interacting with defined binding sites of the framework (Fig. 6).

## 5. Theoretical and computational methods

Across the field of chemistry of materials, the use of multi-scale computational strategies is becoming common, and negative adsorption is no exception. We highlight here how the combination of theoretical chemistry methods at different scales was crucial in forming a holistic understanding of the NGA phenomenon and the materials that display this counter-intuitive property.

In the seminal report of the investigation of NGA in DUT-49, one theoretical and two computational tools were combined.^19^ First, we used calculations at the density functional theory (DFT) level to confirm the structures and identify the energetics of the bistable host phases, in the absence of guest molecules. Second, we performed classical grand canonical Monte Carlo (GCMC) simulations to predict the adsorption capacity of the two host phases using a rigid representation of the framework in each structure. Finally, a theoretical thermodynamic framework was used to rationalize the experimental observations based on the thermodynamic potential of each phase in the osmotic ensemble, accounting for both flexibility and adsorption.^65^ While this methodology laid the basis for a multi-scale simulation strategy, it had important limitations that were addressed in later work.

The structure and energetics of the identified NGA materials, for the entire unit cell performed at the quantum chemical (DFT) level, have high computational cost even for relatively “simple” calculations, such as energy minimizations without taking into account guest molecules. This is due to the complex nature of the DUT-49 isoreticular series which have a large unit cell size (unit cell parameter of 46.6 Å and 1728 atoms for DUT-49, for example). Therefore, computational *ab initio* methods routinely available for other flexible MOFs are not readily applicable to NGA materials. It is however possible to apply DFT to smaller subsystems of the NGA materials, such as the organic ligand building block, in order to characterize their deformation under constraint therefore providing a better understanding of mechanical properties, and screening potential for flexibility.^32^ Another possible use of *ab initio* methods for soft porous crystals (and NGA materials in particular) is to leverage them for the optimization (and validation) of classical potentials with high accuracy.^64,66^ This approach has been applied in the literature to a number of flexible MOFs,^67^ creating a novel generation of force fields that can reproduce with chemical accuracy the supramolecular flexibility as well as its coupling with host–guest interactions. More recently, this approach has been extended to the training of equivariant neural network potentials based on *ab initio* data, either produced on a set of training data or on-the-fly during the dynamics. These machine learning potentials (MLPs) show great promise for the description of the dynamics of complex supramolecular assemblies and materials.^68,69^

Simulations have progressed at the classical level beyond the standard GCMC simulations applied to a rigid framework. The implementation and development of hybrid methods, which combine Monte Carlo and molecular dynamics, was suited to account for both the adsorption and flexibility of the host frameworks. These hybrid approaches, developed for breathing in MIL-53,^70^ used two distinct implementations where either the molecular dynamics ensemble employs fixed volume or pressure. These simulations that use molecular dynamics with fixed pressure were used to provide complementary insight into the molecular origin of the guest-triggered abnormal structural behavior of DUT-49 (Fig. 6).^64^ However, these direct osmotic ensemble simulations (fixed pressure) can be very difficult to converge. Alternatively, by combining many fixed volume hybrid simulations the complete osmotic potential of DUT-49 was constructed.^26^ This detailed landscape explains the experimentally observed NGA transition, reopening, temperature dependence, and even the hysteresis between adsorption and desorption. A complete thermodynamic description of NGA was further developed beyond this atomistic description to model pore structures such as the slit-pore model.^35^ Our general description of responsive adsorption processes in these idealized pore models outlined the key characteristics required for gate-opening and NGA further demonstrating the exact bistable nature necessary for NGA. These classical simulations have allowed us to describe NGA completely at the atomistic level, and even to go beyond, but due to high computational costs in sampling and the production of accurate classical potentials, we have yet to apply this understanding in a large-scale predictive investigation of NGA to find new examples.^71^

At the mesoscopic simulation scale, there is still relatively little known about NGA materials. However, we expect to also find many surprises, for two reasons. Firstly, it was shown on multiple other families of soft porous crystals that specific phenomena or behaviors can emerge at a length scale that is larger than that of the crystal's unit cell, involving for example the coupling between neighboring cells or the external surface of the crystallite. It was demonstrated that parameters such as the crystal size, the textural properties or the chemistry at the external surface are coupled with adsorption-induced flexibility.^72^ To give one example, crystal size can drastically impact the flexibility of MOF Cu_2_(pypz)_2_, and in turn its macroscopic xylene isomer separation efficiency in the liquid phase.^73^ We have highlighted above in Section 3.2 that crystal size and the presence of defects in DUT-49 have an important influence on its negative adsorption properties.^28^ In this area, theoretical modeling was limited until recently to analytical models, or simple numerical simulations relying on lattice-based^74^ or finite elements approaches.^62^ However, recent modeling work published on other soft porous crystals has shown that crystal size effects can be more directly addressed through atomistic simulations, in particular for stimuli-responsive MOFs involving a reversible phase transition.^63^ Moreover, Keupp and Schmid have also demonstrated that atomistic molecular dynamics simulations of nano-sized crystallites of soft porous crystals are within computational reach. They achieved this through the combination of periodic boundary conditions (PBC) removal and free energy sampling with a distance restraint.^75^ This method paves the way to the molecular simulation of finite-size effects. However, providing a chemically accurate description of the external surfaces of MOF crystals remains a widely open challenge.

Finally, while the thermodynamics of NGA have been thoroughly established (as described above), a review of the literature published on flexible MOFs shows that they exhibit a rich diversity of behavior when guest adsorption is coupled to external stimuli, of a physical or chemical nature.^76^ In particular, it would be of interest to study the coupling of NGA with temperature, but also more generally to mechanical pressure, anisotropic stress, and gas mixture co-adsorption. It is even possible to couple NGA with photoresponsive azobenzene ligands, to create a light-switchable NGA material, *via* the buckling of the linkers.^8^ We can therefore envision the development of computational methodologies that would allow for the screening of materials databases for an application like this one and, going even further, to perform a high-throughput screening of materials to rank possibility of specific couplings between chosen, arbitrary stimuli. This appears for now to be an ambitious challenge, but through better understanding of the key physical and chemical characteristics of building units required for each function, it could become possible, just like it is possible today to screen materials databases for adsorption capacity, gas separation selectivity, optical band gap, or mechanical toughness.

## 6. Design of new NGA materials, mechanisms and emerging applications

So far we have reported 7 MOF materials that show NGA. All of them are based on the DUT-49-type topology (**fcu-a**) and can be considered isoreticular DUT-49 derivatives. Beyond these examples no other examples of NGA that follow the mechanism described above have yet been reported (Table 1).

**Table 1:** Summary for the reported NGA frameworks and corresponding NGA conditions

MOF	NGA conditions, guest (temperature)	Ref.
DUT-49	N_2_ (77 K), Ar (77–100 K), Kr (120–150 K), Xe (175–210 K), CO_2_ (230–240 K), CH_4_ (90–130 K), C_2_H_6_ (200–240 K), C_2_H_4_ (169–199 K), C_3_H_8_ (261 K), *n*-C_4_H_10_ (288–308 K), C_4_H_6_ (298–303 K)	19, 27, 29 and 52
DUT-50	N_2_ (77 K), Ar (87 K), CO_2_ (230–240 K)	32
DUT-140	CH_4_ (111 K)	77
DUT-148	N_2_ (77 K), CH_4_ (111 K), *n*-C_4_H_10_ (298 K)	30
DUT-160	N_2_ (77 K), CH_4_ (111 K)	30
DUT-161	N_2_ (77 K)	30
DUT-163	CH_4_ (111 K), *n*-C_4_H_10_ (298 K)	8

However, the series of DUT-49-related solids provides a clear picture of features that are required for NGA materials: high porosity, a relatively soft framework and the ability to undergo large scale reversible pore contraction. Beyond that the presence of mesoporosity seems to be a crucial factor that governs NGA at least in the DUT-49 topology.

Realistically, there might be other materials with properties very different to DUT-49 that can show NGA. In 1963 Riekert reported adsorption isotherms (CO_2_ at 195 K, ethane at 195 K) of synthetic HY zeolite exhibiting features resembling to a certain extent NGA in DUT-49.^78^ Unfortunately, the work contains no structural analysis but discusses a potential thermodynamic explanation that is strikingly similar to the mechanism established for NGA in DUT-49. This illustrates that new microporous NGA materials could also be feasible if one engineers a kinetic barrier associated with a structural transformation mechanism coupled to gas uptake going far beyond mesoporous MOFs. Predicting metastable states in porous solids, in particular during the adsorption process, remains a challenge from a theoretical point of view. Imagination and creative design of materials deliberately tuned towards certain activation barriers may open new opportunities for the discovery of useful phenomena far away from thermodynamic equilibrium.^11^

A different approach to trigger gas release *via* structural contraction under isothermal/isobaric conditions akin to NGA is the utilization of a secondary stimulus. A wide range of photoresponsive frameworks are known to exhibit a reduction in uptake upon application of a light-source.^10,79^ However, in the vast majority of cases the reduction in uptake (negative slope in the isotherm) is not associated with large-scale pore deformation but rather internal heating *via* light-absorption and correlated non-radiative relaxation processes.^80^ However, we recently reported that adsorption-driven structural contraction can be initiated in the DUT-49-type MOF (DUT-163) with an azobenzene backbone.^8^ Upon adsorption of iso-butane at 300 K and parallel application of 365 nm irradiation, DUT-163 was found to contract as a result of the simultaneous application of light-gas stimulation.

This mechanism can be understood as follows: the energy (thermodynamic driving force) for structural contraction is provided by the gas adsorption, and the barrier for contraction is overcome (kinetic trigger) by light activation which changes the chemistry of the linker backbone. This mechanism allows for spatio-temporal control of contraction and subsequent gas release.

Beyond curiosity and fascination, the counterintuitive phenomenon of self-amplification (NGA/PA) may trigger novel applications for damping, actuation, separation, pneumatic systems, robotics, pressure management and control. To date, little has been explored in the utilization of NGA and the field is at its earliest beginning, mainly because only few materials have been reported demonstrating NGA and admittedly their operation is not practical yet. However, the ability to manipulate dosing gas pressures actively in a range from kPa to several 100 kPa, in a wide temperature range and with various gases, is remarkable. Such stimuli responsive PA materials could play an important role in dampers or safety systems, in which gas ejection is required as soon as a certain critical pressure is achieved. In pneumatic systems a pressure pulse may be required to switch a valve. In autonomous robotic architectures pneumatic systems are used as an alternative to electronic motors. PA materials could play an important role for the pressure regulation or the realization of oscillating motors propelled by moderate pressure. *Vice versa*, internal motors remotely triggered could lead to PA materials powered by external control systems.

To achieve this next generation, PA materials require a higher degree of robustness and system integration as monolithic materials or thin films. An ideal family may be dynamic Zr-MOFs, COFs (COF - covalent organic framework) or PAFs (PAF - porous aromatic framework) as they are highly tolerant towards chemicals. However, to date, the number of 3D COFs is still limited and mesoporous dynamic systems have not been widely explored.

Gas separation using PA materials is another unexplored field. The sudden release of one component, contracting the framework, could provide beneficial aspects in terms of sorbent regeneration and heat management. Moreover, an uncommon selectivity may be expected if one component stimulates the contraction being preferentially adsorbed in the micropore of the forming cp phase while the second component is pushed out as a reversed mechanism compared to molecular sieving. An obvious example would be CO_2_/CH_4_ separation, which is also industrially relevant, but also O_2_/N_2_, ethane/ethane and many more.

Today, the deliberate engineering of metastable porous systems is in its infancy. It is to be expected that a much wider variety of phenomena such as positive desorption can be explored also technologically if such barriers and their deliberate engineering can be better understood from a materials and adsorption point of view. The rapid advancement of digitalization, computational methods and machine learning may advance the field beyond serendipitous discoveries and enable rationally designed applications and materials in the future.

## 7. Conclusions

NGA is a novel and counterintuitive yet generalizable phenomenon currently observed for a limited number of mesoporous MOFs with the ability to sustain breathing transitions. A subtle interplay of pore size, framework softness, but also temperature and adsorption stress exerted by the adsorbate, controls the structural contraction process and occurrence *vs.* absence of NGA. The solid phase transition of the MOFs and its coupling to the liquid phase nucleation inside the pores are the origin of energetic barriers leading to long-lived metastable states during the adsorption process inducing NGA. Computational methods nowadays provide a complete energetic landscape of the fluid-solid system providing means for rationalization. The deliberate design of model materials varying in composition, pore size, mechanical properties, particle size *etc.* leads to empirical guidelines for the discovery of a wider range of PA materials. NGA is certainly not exclusive to MOFs. However, the crystalline structure and reduction in pore wall thickness combined with the increase in porosity and reduction in mechanical robustness render MOFs ideal materials capable of responding to intermediate and high adsorption-stress with pronounced structural deformations. Following recent development in crystalline and highly porous COFs, PAFs, zeolites and other crystalline porous materials it would be no surprise to see NGA phenomena in porous materials other than MOFs. Beyond the discovery of novel NGA materials, it will be interesting to find novel strategies to analyze the complex energy landscape of dynamic porous solids as they allow us not only to identify the mechanism but also potentially new phenomena. Triggering the adsorption-induced contraction of highly porous systems based on metastable states *via* orthogonal external stimuli such as light, electric or magnetic pulses, or even simply thermally, may lead to remote controlled dynamic solids offering a wide range of functionality in the future.

## Author contributions

S. Ka. conceptualized and supervised the project. All authors contributed to writing and revising the manuscript.

## Data availability

The data that support the findings of this study are available from the corresponding author upon request.

## Conflicts of interest

There are no conflicts to declare.
